# Predictors of persistent organ failure in ICU patients with severe acute pancreatitis

**DOI:** 10.1186/cc12345

**Published:** 2013-03-19

**Authors:** I Aleksandrova, M Ilynskiy, S Rei, V Kiselev, G Berdnikov, L Marchenkova

**Affiliations:** 1 Hospital Research Institute for Emergency Medicine N.V. Sklifosovsky, Moscow, Russia

## Introduction

Severe acute pancreatitis (SAP) requiring admission to an ICU is associated with high mortality (hospital mortality reached 42%) and long lengths of stay [1]. Survival among patients with predicted SAP at admission has been shown to correlate with the duration of organ failure (OF) [2]. The systemic determinant of severity in a new classification of acute pancreatitis (AP) is also based on identification of patients with transient or persistent OF [3].

## Methods

The aim of the study was to retrospectively determine the predictors of early persistent OF in ICU patients with SAP. The analysis involved 152 patients. The median time interval between the onset of AP and admission was 24 (9; 48) hours. The patients were divided into two groups: the first group (*n *= 46) had transient OF and the second group (*n *= 106) had persistent OF. The ability of the APACHE II score, total SOFA score and number of organ/system failure to discriminate transient from persistent OF was explored with receiver operating characteristic (ROC) curves.

## Results

Hospital mortality was significantly higher in the second group as compared with the first group (45% vs. 7%, *P *= 0.000); while infectious complications were 39% versus 11% (*P *= 0.001) and median lengths of ICU stay were 8 (5; 18) days for the second group and 6 (4; 7) days for the first group (*P *= 0.001). Optimum cutoff levels (by ROC curve analysis) were APACHE II score ≥12 (sensitivity 0.790; 1 - specificity 0.119), total SOFA score ≥4 (sensitivity 0.829; 1 - specificity 0.190), and failure ≥2 organs/systems (sensitivity 0.819; 1 - specificity 0.214). See Table 1.

**Table 1:** 

Variable	AUC	Standard error	95% CI
APACHE II score	0.934	0.023	0.889 to 0.980
SOFA score	0.908	0.026	0.857 to 0.960
Number OF	0.901	0.026	0.849 to 0.952

AUC, areas under the curves.

## Conclusion

Persistent OF in ICU patients with SAP is associated with the highest risk of hospital mortality. Early identification of patients with a high likelihood of persistent OF (APACHE II score ≥12, total SOFA score ≥4, failure ≥2 organs/systems) is an important goal in determining optimal management of ICU patients with SAP.
